# Magnetization reversal and microstructure in polycrystalline Fe_50_Pd_50_ dot arrays by self-assembling of polystyrene nanospheres

**DOI:** 10.1080/14686996.2016.1201414

**Published:** 2016-08-10

**Authors:** Paola Tiberto, Federica Celegato, Gabriele Barrera, Marco Coisson, Franco Vinai, Paola Rizzi

**Affiliations:** ^a^ Nanoscience and Materials Division, INRIM, Torino, Italy; ^b^ Chemistry Department, Università di Torino, Torino, Italy

**Keywords:** Self-assembling, magnetic thin film, magnetization reversal, microstructure, phase transformations, 40 Optical, magnetic and electronic device materials, 101 Self-assembly / Self-organized materials, 106 Metallic materials, 203 Magnetics / Spintronics / Superconductors, 302 Crystallization / Heat treatment / Crystal growth, 503 TEM, STEM, SEM

## Abstract

Nanoscale magnetic materials are the basis of emerging technologies to develop novel magnetoelectronic devices. Self-assembly of polystyrene nanospheres is here used to generate 2D hexagonal dot arrays on Fe_50_Pd_50_ thin films. This simple technique allows a wide-area patterning of a magnetic thin film. The role of disorder on functional magnetic properties with respect to conventional lithographic techniques is studied. Structural and magnetic characteristics have been investigated in arrays having different geometry (i.e. dot diameters, inter-dot distances and thickness). The interplay among microstructure and magnetization reversal is discussed. Magnetic measurements reveal a vortex domain configuration in all as-prepared films. The original domain structure changes drastically upon thermal annealing performed to promote the transformation of disordered A1 phase into the ordered, tetragonal L1_0_ phase. First-order reversal magnetization curves have been measured to rule out the role of magnetic interaction among crystalline phases characterized by different magnetic coercivity.

## Graphical abstract

**Figure:**
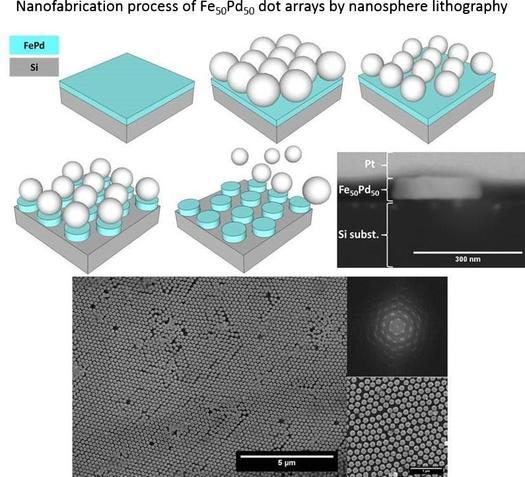

## 1. Introduction

Development of new materials for applications responding at the need for continuous optimization of performances, energy saving and environment protection is one of the present challenges in material science and is sustained by advances in nanotechnology.[1–4] Reduced dimensions, aspect ratio and large surface areas indicate that patterned nanostructures may be good candidates for applications in sensors, biomedical chips, and magnetically assisted drug carriers.[5–7] In this quest for nanoscale, novel materials with multi-functional magnetic properties, nano-elements may be fabricated by controlling shape and size. In addition, the ability to position each element at a desired location and to organize into two- or three-dimensional architectures further improves the control of functional properties (i.e. magnetic response).[8,9] Many nanodevices with unique magnetic properties are constituted by arrays of nanoelements, where averaged properties obtained by collective measurements (e.g. Vibrating sample magnetometry (VSM) or superconducting quantum interference device (SQUID) magnetometry) are not sufficient to get a deeper insight into the magnetization process. Such nanomagnets typically exhibit complex magnetization reversal due to the fact that their dimension is comparable to the magnetic domain wall widths. An example is given by nanomagnets constituted by arrays of nanodisks, where magnetization reversal strongly depends on dot aspect ratio passing through several configurations, evolving from a single domain towards a more complex one as vortex formation.[10]

Nanolithography techniques are mostly used to design nano-elements [11]. Optical lithography and sequential nanolithographic techniques (electron-beam, X-ray, and focused ion beam) have limited use in industrial environments due to their low-performance and high cost. As a consequence, new nanofabrication processes characterized by high throughputs and yields represent key challenges for the large-scale, inexpensive production of nanodevices. The search for alternatives to the current limited technologies has led to a growing interest in preparing templates/masks for patterning [12] for controlled growth of nanostructures (i.e. nanoporous materials [13]). A lithography-free technique to attain self-assembled and highly ordered porous surfaces with nano-size dimensions would immediately offer new possibilities for their technological application. Self-assembling methods provide new routes for the large-scale and inexpensive production of nanodevices. This technique is widely used to design masks for patterning nanostructures on thin films surface by using polymeric spheres (i.e. polystyrene nanospheres and di-block copolymers) [14,15] and has been shown to be effective in designing dot arrays on several magnetic thin films.[16,17] Arrays of nanodots having chemical compositions characterized by high magnetic anisotropy values (i.e. FePt and FePd alloys) have been the subject of intensive research due to their application in information storage devices (i.e. hard disks and magnetoresistive random-access memory).[18,19] In such binary systems, the fraction of high anisotropy, ordered-L1_0_ to disordered-A1 phase alloys strongly depends on film stoichiometry and thickness given the growth parameters (i.e. substrate, deposition temperature, deposition rate, and pressure).[20,21] Subsequent thermal treatments have been shown to increase the magnetic anisotropy value by completing the A1 to L1_0_ phase transformation.[22,23] In order to assess if large-area patterning techniques can represent a valid alternative to electron-beam nanolithography, a careful balance among viability, intrinsic lattice disorder due to self-arrangement, and its effect on functional magnetic properties has yet to be fully investigated. Fe_50_Pd_50_ dot arrays having different aspect ratio and inter-dot distance have been produced by polystyrene nanosphere lithography on sputtered thin films. Such a composition deals with different magnetization mechanisms, being vortex nucleation in the as-prepared state and the one typical of high-anisotropy phase (as L10 phase), by simultaneously tuning crystallization process and patterning geometry. To this aim, magnetization reversal has been carefully studied by magnetization curves and magnetic force microscopy and explained taking into account the nanodot microstructure.

## 2. Experimental details

**Figure 1.**
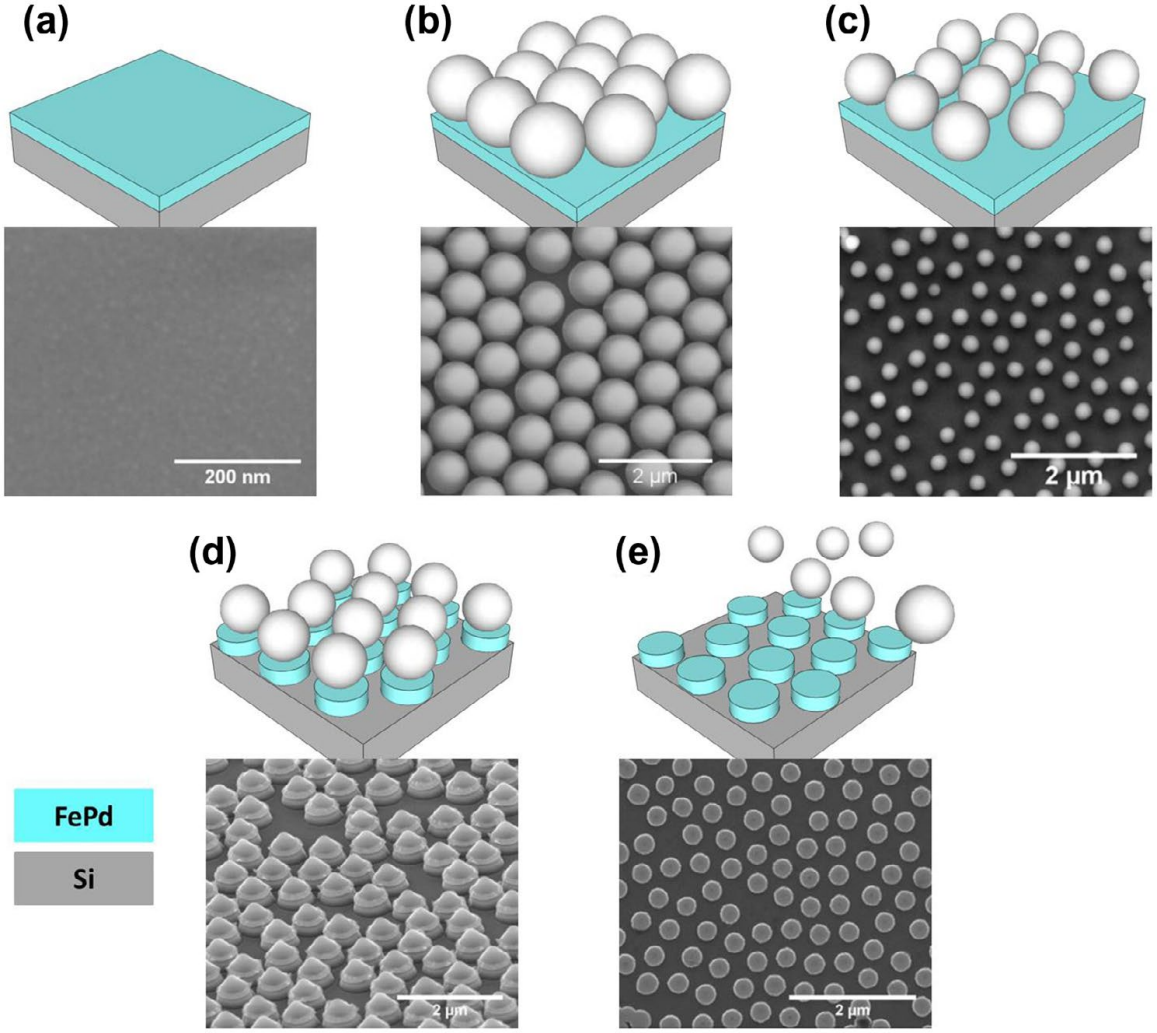
Schematic description of the nanofabrication process together with corresponding SEM images: (a) sputter deposition of a Fe_50_Pd_50_ layer on a Si substrate; (b) deposition of a monolayer of polystyrene nanosphere; (c) plasma etching in Ar^+^ to reduce PN diameter; (d) sputter etching to remove magnetic layer among spheres; (e) sphere removal by sonication.

Arrays of Fe_50_Pd_50_ dot nanostructures are fabricated by exploiting polystyrene nanosphere (PN) lithography. The multi-steps process is schematically illustrated in Figure 1 and each step is described together with corresponding sample morphology acquired by scanning electron microscopy (SEM).

**Figure 2.**
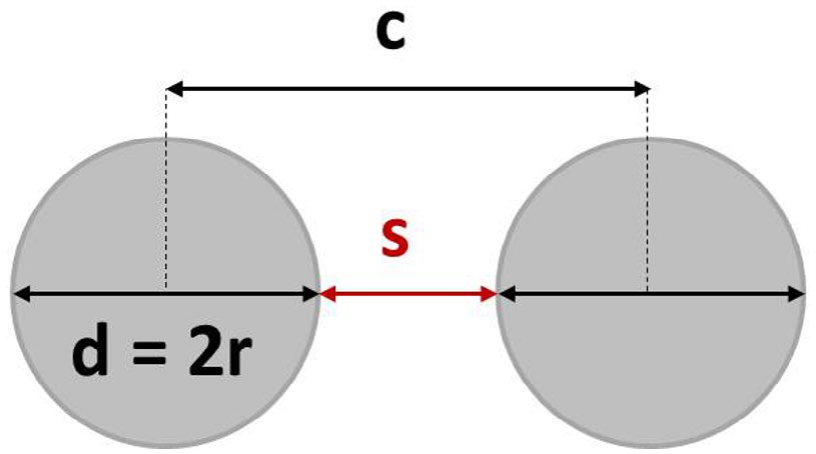
Scheme of patterned dots reporting diameter *d*, interdot distance *s*, center-to-center distance *c*.

In the first step shown in Figure 1(a), a continuous Fe_50_Pd_50_ layer (thickness *t* = 50 and 35 nm) is deposited by rf-sputtering on a Si substrate (orientation [100]) covered with a native oxide layer. The as-deposited film consists of a polycrystalline fcc Fe_50_Pd_50_ disordered solid solution, being sputtered without heating the substrate.[21] Subsequently, a monolayer of commercially available polystyrene nanospheres (PN, starting diameter 220 and 500 nm) is deposited by floating technique onto the magnetic thin film. The PNs undergo a self-assembling process that leads to the formation of a monodisperse hexagonally close-packed lattice (see Figure 1(b)). In the third step shown in Figure 1(c), in order to obtain an array of hexagonally arranged isolated dots, the PN diameter is reduced by plasma etching in Ar^+^. The final dot diameter can be tailored as desired by setting the etching time. At this stage, polystyrene nanospheres are used as a hard mask for sputter etching with Ar^+^ ions to remove the remaining magnetic material among the PNs, as shown in Figure 1(d). In the last step, the PNs are removed by sonication in deionized water, finally obtaining an array of Fe_50_Pd_50_ dots having the same composition and thickness as the starting continuous thin film (Figure 1(e)). The corresponding SEM image shows the final well-ordered array of Fe_50_Pd_50_ dots, though the self-assembly process naturally produces some defects in the nanospheres array (i.e. vacancies, dislocations and distribution of PN diameters) [24] that are transferred to the final Fe_50_Pd_50_ dot array. Selected samples of Fe_50_Pd_50_ dot arrays having different diameters (*d* = 330 and 160 nm) and film thickness (*t* = 50 and 35 nm) have been prepared following the process sketched in Figure 1. Center-to-center distance (*c* = 500 and 220 nm) is fixed by the initial PN diameter. The aspect ratio *β* is defined as the ratio between film thickness *t* and dot diameter *d*. Our arrays are characterized by *β* values ranging from 0.11 to 0.32. A scheme of the patterned structure reporting the relevant geometric parameters is shown in Figure 2.

The structural characterization of continuous thin films and dot samples was performed by scanning electron microscopy (SEM) and scanning transmission electron microscopy (STEM). Transmission electron microscopy (TEM) and high resolution TEM (HRTEM) analysis was performed on the cross section of the annealed samples (both continuous film and dotted samples) by preparing lamellae thinned by focused ion beam (FIB).[25]

All as-prepared and annealed samples have been magnetically characterized at room temperature by means of a high-sensitive alternating gradient field magnetometer (AGFM). Room temperature hysteresis loops have been measured in the field range –18 kOe < *H* < 18 kOe in the longitudinal and perpendicular configuration. In order to achieve a more detailed description of the magnetization process in the dot arrays, first order reversal curves (FORC) [26] have been measured and exploited as a powerful tool in the characterization of magnetic systems presenting different magnetic phases.[27–29] Such a technique has been already successfully employed to study the coexistence of different magnetization reversal modes in magnetic nanodot arrays,[30,31] on arrays of nanowires and on other magnetic systems.[32] To this aim, first order reversal curves with the magnetic field applied in the film plane have been measured to get insight on distribution of coercivity and interaction of the dots, and on the portion of reversible and irreversible magnetization reversal processes that are present in the system. FORC diagrams have been extrapolated from FORC experimental curves by an ad hoc developed analysis procedure. Magnetic force microscopy (MFM) has been performed in lift-mode to image magnetic domain patterns using a commercial tip coated with a ferromagnetic CoCr alloy (MESP-HR, coercive field ≈ 900 Oe). Images have been acquired for samples at perpendicular magnetization remanence and under the application of an in-plane magnetic field.

Highly ordered L1_0_ tetragonal phase can be obtained by growing the film on a heated substrate.[22] However, the high-anisotropy tetragonal phase development can also be promoted by post-deposition thermal treatments. As a consequence, to promote the order-disorder transformation towards the L1_0_-ordered tetragonal phase, selected films have been submitted to furnace annealing in vacuum (base pressure 3 × 10^−5^ mbar) at temperature *T*_a_ = 600°C for time *t* = 1200 s.[21]

## 3. Results and discussion

### 3.1. Structural characterization

**Figure 3.**
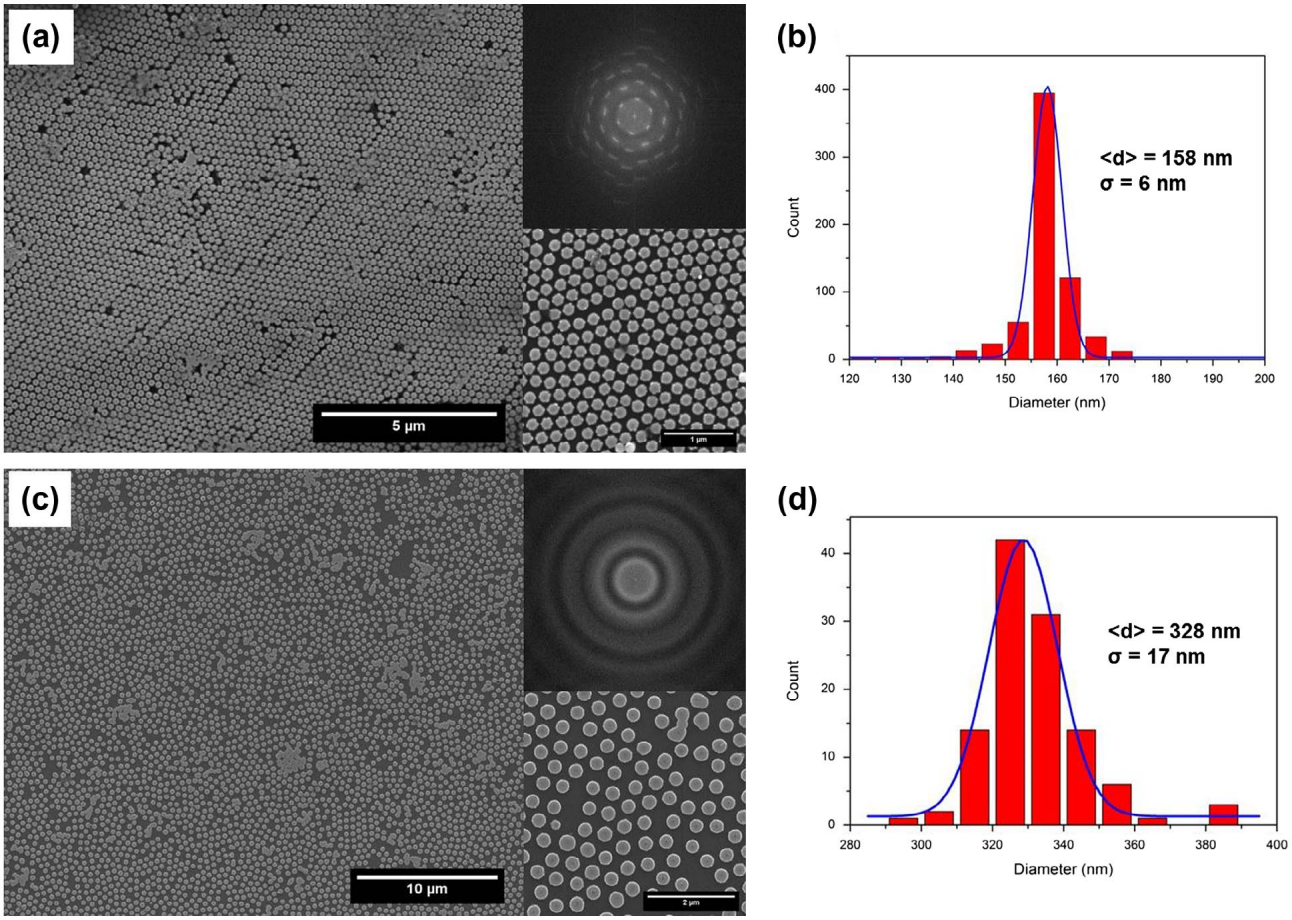
(a) Large-area SEM image of nanodot array (initial NP diameter 220 nm) together with a magnified area (bottom) and a calculated FFT of the image (top); (b) distribution of nanodot diameter to evaluate mean diameter as derived from the image shown in (a). Blue lines: Gaussian fit of the dot size distribution; (c) same as in (a) for a starting NP diameter of 500 nm; (d) same as in (b) but for the image reported in (c).

The results of the patterning process obtained by using polystyrene nanospheres having initial diameter about 220 and 500 nm are visible in Figure 3(a) and (c), respectively, where SEM corresponding images are reported. In both samples, a very wide region of film surface is shown to confirm that the self-assembling technique is effective in patterning the array of dots homogeneously dispersed on the surface, well separated and having a round shape.

The dot array shown in Figure 3(a) is characterized by a center-to-center distance (220 nm) fixed by the initial NP diameter. The distribution of dot diameters, derived by SEM analysis and leading to an average diameter of about 158 ± 6 nm, is reported in Figure 3(b). The distribution is quite narrow and well fitted by a Gaussian bell (continuous line in Figure 3(b)). A magnification of the dot arrays in the SEM image of Figure 3(a) is shown (right, bottom panel) and confirms that the dots are well separated and rounded in shape. The degree of ordering was evaluated by fast Fourier transformation (FFT) and the corresponding 2D FFT image of the array shown in Figure 3(a) (right, top panel) revealed a well-ordered hexagonal diffraction pattern. This indicates the presence of macroscopic close-packed ordered domains with well-defined boundaries and large dimension univocally determined by the self-assembling process.

Dot arrays displayed in Figure 3(c) are synthesized by using PNs having 500 nm initial diameter and consequently a center-to-center distance of 500 nm. Again, diameter distribution obtained by SEM image analysis is shown in Figure 3(d) and points to an average diameter of about 328 ± 17 nm. For higher NP diameters, the distribution is broader than that shown in Figure 3(b) and still fitted by a Gaussian bell. The 2D FFT image reported (top right panel in Figure 3(c)) points to a loss of order as indicated by a the presence of intensity rings caused by a diffused scattering. This reduction of order leads to a decrease of crystallographic domain size evolving to a glasslike structure, as also visible in the corresponding SEM image (Figure 3(c)).

The loss of well-ordered hexagonal lattice, in the sample obtained by 500 nm diameter spheres with respect to that obtained by spheres with lower diameter, can probably be ascribed to a different chemical functionalization of the spheres surface that modifies the interaction forces acting during the self-assembling process and thus controlling the degree of ordering.[33]

**Figure 4.**
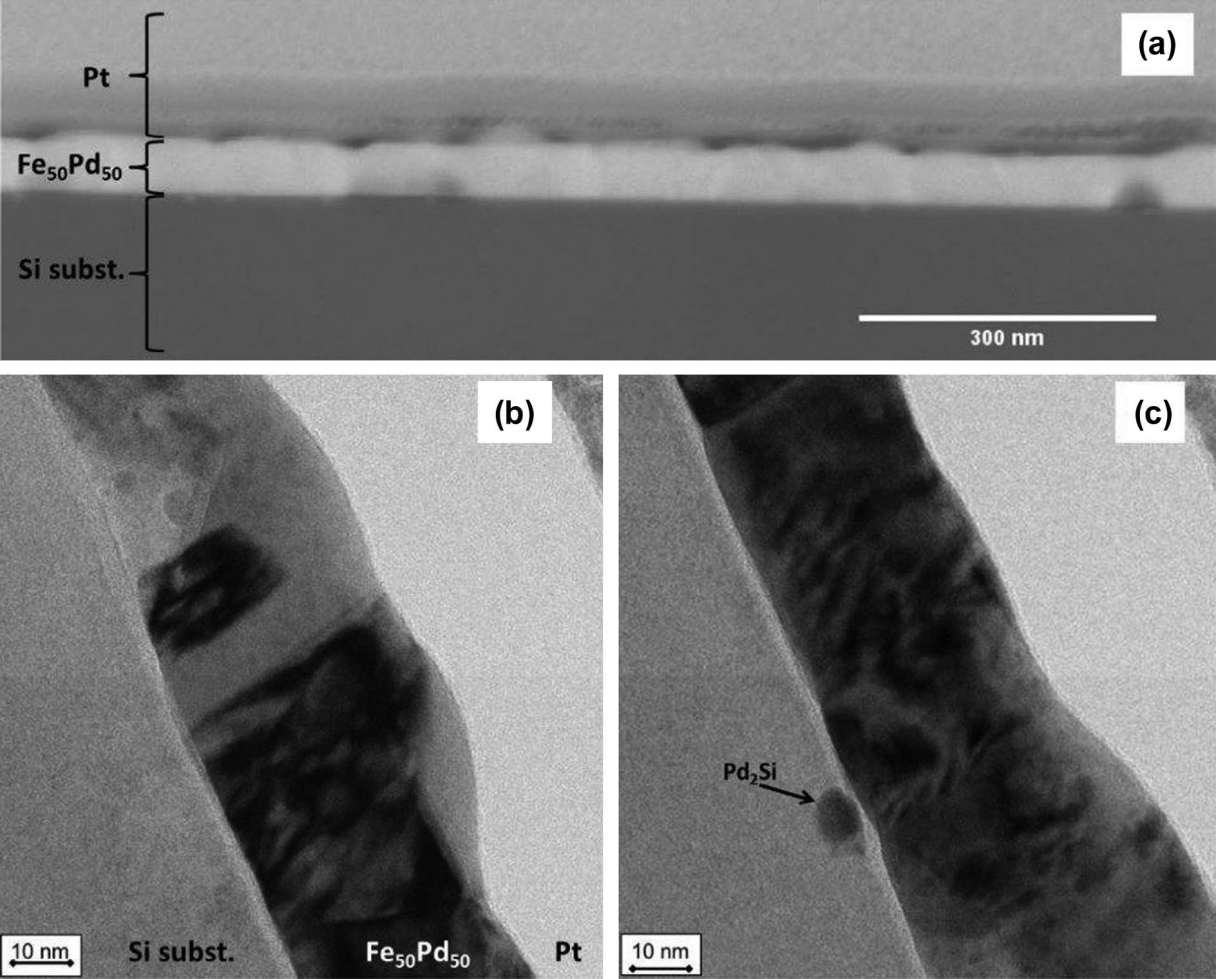
STEM (a) and bright field TEM (b) images of cross section of continuous Fe_50_Pd_50_ (thickness 50 nm) annealed at *T*_a_ = 600°C for 1200 s. In (b) Pd_2_Si and L1_0_ ordered phase are present.

A structural characterization of the continuous thin films prior to the patterning process was performed to check their microstructure. X-ray diffraction (XRD) analysis performed at grazing angle for the as deposited continuous FePd film shows the presence of a disordered γ-FePd phase characterized by a 〈111〉 texture.[21] After annealing, the disordered structure disappears and peaks related to the ordered L1_0_-Fe_50_Pd_50_ tetragonal phase are observed beside a peak that can be attributed to the Pd_2_Si phase. Textures are still evident in the L1_0_-Fe_50_Pd_50_. In addition, SEM analysis reveals the presence, on the annealed sample, of areas a few micrometers large composed by needle-like and rounded crystals. Elemental maps performed in these areas reveal the presence of palladium silicides (Pd_2_Si) and crystals that can be attributed to α-Fe.[21] In fact, the formation of Pd_2_Si during annealing depletes the film in Pd increasing the Fe content to amount greater than 50%at., at which, according to the Pd-Fe phase diagram, α-Fe and FePd are in thermodynamic equilibrium.[34] The cross section of the continuous sample after annealing observed by STEM (Figure 4(a)) shows a roughness on the surface probably due to the change in structure from disordered fcc to ordered tetragonal phase. The film thickness is observed to vary from 40 nm to 65 nm. TEM images of the annealed continuous film are shown in Figure 4(b) and (c). The presence of the L1_0_ ordered phase, characterized by columnar grains with diameter below 30 nm, is confirmed as visible in Figure 4(c). In addition, the formation of rounded Pd_2_Si crystals below 50 nm size is also observed by TEM image reported in Figure 4(b) in the Si substrate immediately under the thin film.

**Figure 5.**
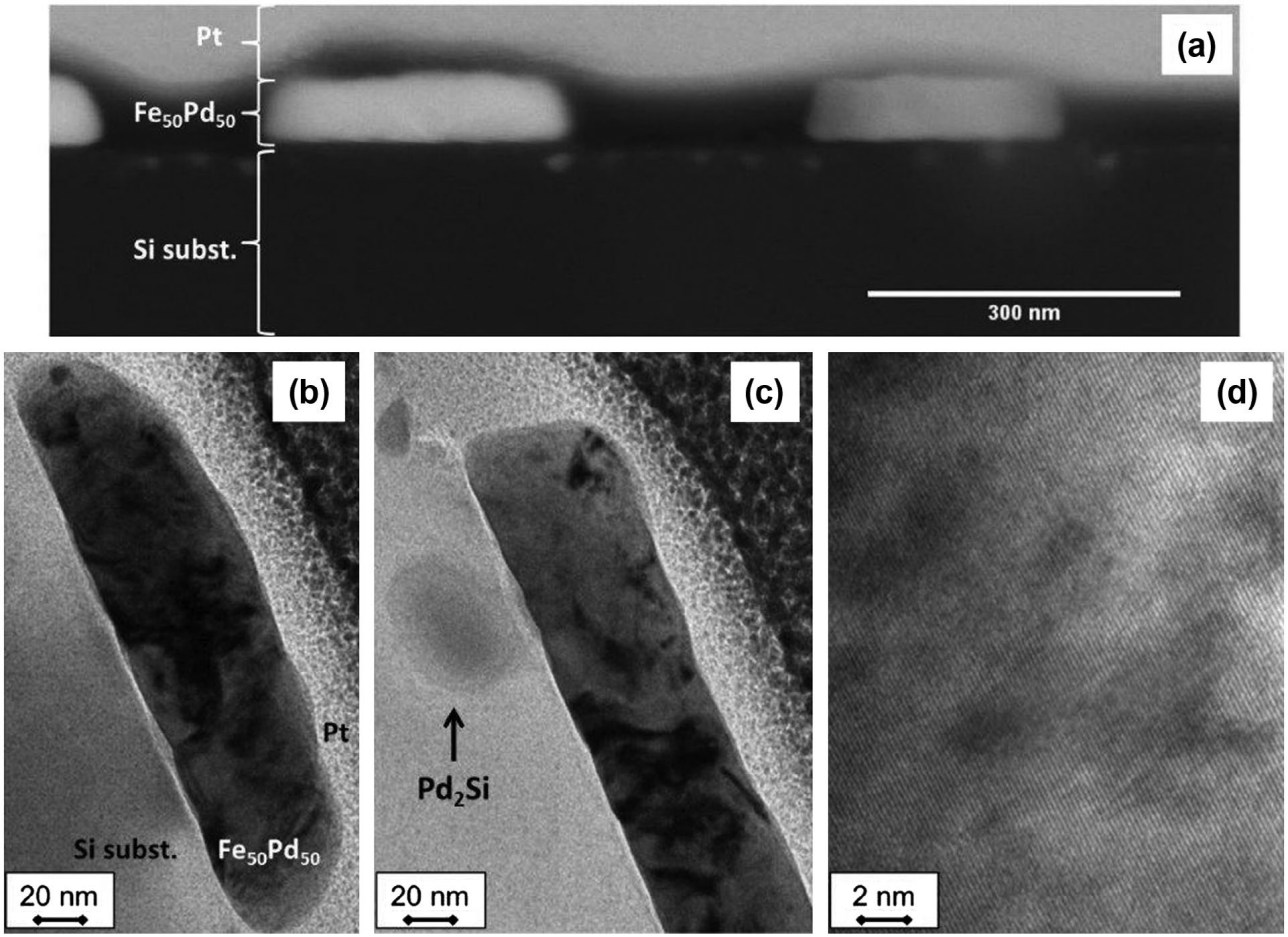
(a) STEM; (b) and (c) bright field TEM; (d) HRTEM images of cross section of patterned Fe_50_Pd_50_ thin film (dot diameter 328 nm and thickness 50 nm) annealed at *T*_a_ = 600°C for 1200 s. In (b), (c) the L1_0_ ordered tetragonal phase is present, while a Pd_2_Si grain can be observed in (c). A magnified view of the dot from panel (c) is shown in (d).

Cross-sectional STEM of the annealed dot sample (*d* = 328 nm and *t* = 50 nm) reveals a homogeneous morphology of the dots (see image in Figure 5(a)). The length of dots, observed in STEM image, are different. In particular, the dot on the right-hand side appears to be smaller than 328 nm (its nominal diameter) because the sample cross section obtained by FIB does not exactly cut the patterned sample along the dot diameter.

TEM bright field images of the same cross section reported in Figure 5(b) confirm that dots have a disk-like shape evidencing the same microstructure of the annealed continuous thin film. The Pd_2_Si phase is sometimes observed by TEM in the Si substrate under the dots (Figure 5(c)). In Figure 5(d) an HRTEM image is reported and confirms the presence of a crystalline phase, presumably the L1_0_ ordered tetragonal phase as indicated by XRD patterns.[21]

### 3.2. Magnetic characterization

**Figure 6.**
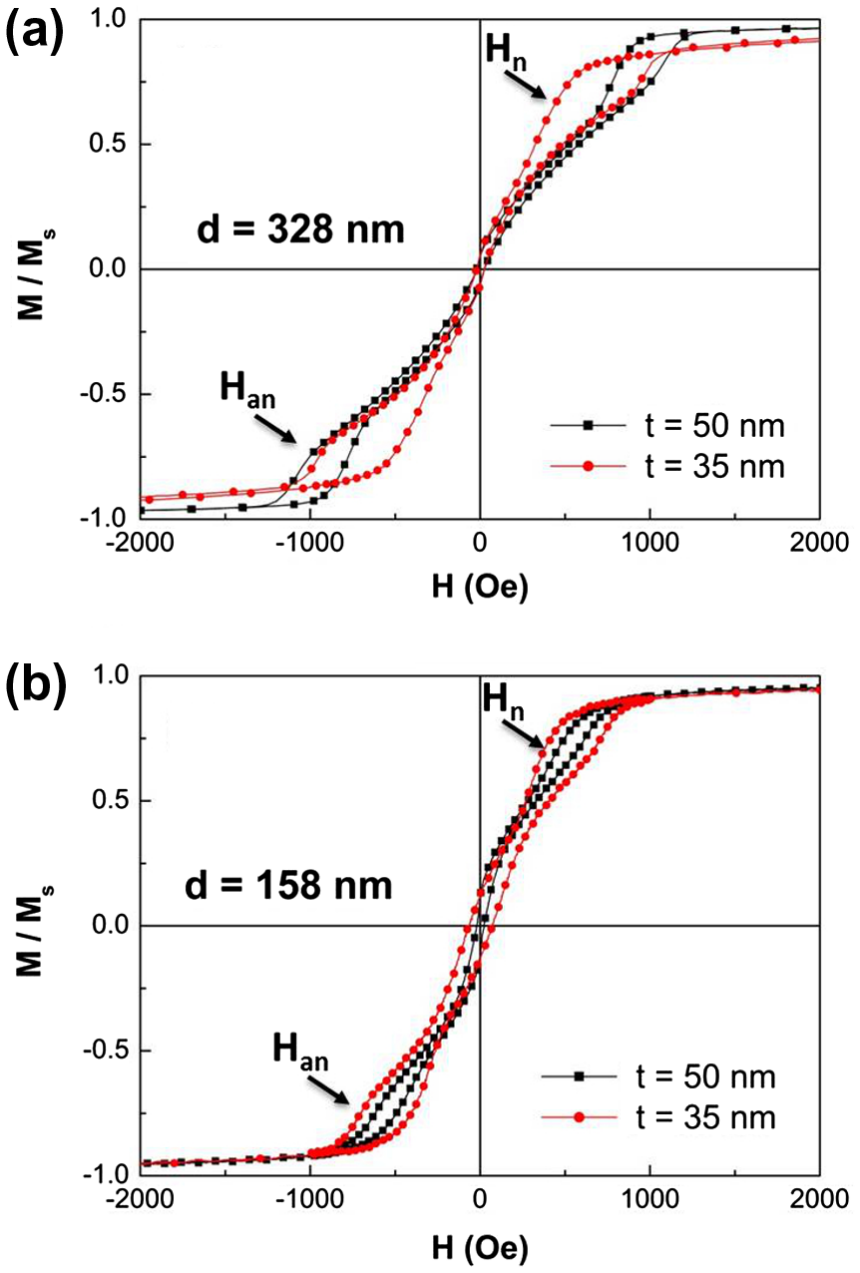
(a) Room-temperature hysteresis loops of Fe_50_Pd_50_ dot arrays in the as-deposited condition, having different thickness (square: 50 nm and circles: 35 nm) and having mean diameter of 328 nm; (b) same as in (a) with dot mean diameter of 158 nm.

Figure 6 shows a set of normalized room-temperature hysteresis loops of Fe_50_Pd_50_ dot arrays belonging to the four families, in the as-deposited condition (*d* = 328 and 158 nm, *t* = 50 and 35 nm, *β* ranging from 0.11 to 0.32, *c* = 500 and 220 nm). The magnetic field was applied in the film plane and parallel to the Fe_50_Pd_50_ dot surface.

The magnetization reversal of dot array with *d* = 328 nm and center-to-center distance *c* = 500 nm (Figure 6(a)) is typical of a magnetic vortex behavior.[10] When the applied magnetic field is reduced from saturation to the nucleation field *H*_n_, a rapid magnetization jump appears, indicating the nucleation of a single magnetic vortex inside each dot. The low-field reversible linear part of the hysteresis loops corresponds to the movement of vortex core perpendicular to the applied field across the dot. When the applied magnetic field is further reduced to reach the annihilation field *H*_an_, the magnetization jumps again and the magnetic vortex is expelled.

By simultaneously reducing the dot diameter and the center-to-center distance (*d* = 158 nm and *c* = 220 nm respectively), the fingerprint of the magnetization behavior by vortex nucleation is always visible in Figure 6(b). However, higher values of coercive field and magnetization remanence appear in the hysteresis loops, especially for the film having *t* = 35 nm (*β* = 0.22). This behavior can be accounted for by two simultaneous effects: (1) the reduction of geometrical dimensions and (2) a dot diameter distribution close to the critical diameter value to reverse magnetization by vortex mechanism (see Figure 3(b)). As a consequence, some dots reverse their magnetization coherently in a single irreversible jump, without developing an intermediate vortex state as the majority of the dots in the array single magnetization state reversal.[10]

The values of *H*_n_, *H*_an_, and the slope of the linear part of hysteresis loops are strongly dependent on geometrical parameters as dot diameter (*d*), thickness (*t*) and on center-to-center distance (*c*).[10] The parameters corresponding to the curves reported in Figure 6(a) and (b) are summarized in Table 1. As it can be observed in Figure 6(a), dotted samples with *d* = 328 nm and *c* = 500 nm are simultaneously characterized by a lower nucleation ad annihilation field with decreasing the dot thickness (*t*), and a higher value of initial susceptibility *χ*_0_ calculated from the reversible part of hysteresis loops (slope of the magnetization curve around zero applied field). This behavior is in agreement with previous experimental and theoretical results obtained in regular dot arrays obtained by electron beam lithography having similar compositions.[35]

**Table 1.** Dot diameter (*d*); film thickness (*t*); center-to-center distance (c); nucleation field (*H*_n_); annihilation field (*H*_an_) and initial susceptibility (*χ*_0_) of all Fe_50_ Pd_50_ as deposited patterned films.

*d* (nm)	*t* (nm)	c (nm)	*H*_n_ (Oe)	*H*_an_ (Oe)	*χ*_0_
328	50	500	803	−1097	1.0 × 10^−7^
328	35	500	319	−965	2.3 × 10^−7^
158	50	220	483	−605	2.8 × 10^−7^
158	35	220	338	−736	5.8 × 10^−8^

In our opinion, the magnetostatic interactions among dots can be neglected in dotted samples having diameter *d* = 328 nm and center-to-center distance *c* = 500 nm, considering that the distance *s* between each dot is higher (around 180 nm) than the radius (see Figure 2).[36] On the contrary, the smaller dots (*d* = 158 nm and *c* = 220 nm) having distances between each dot smaller than the dot radius (around 60 nm) may be characterized by weak magnetostatic interactions and may affect the values of *H*_n_ and *H*_an_.[36]

To study the effect on the magnetic properties of the transformation kinetics from A1 fcc disordered FePd to the L1_0_ tetragonal phase, furnace annealing at temperature *T*_a_ = 600°C for 1200 s in vacuum has been performed.[21]

**Figure 7.**
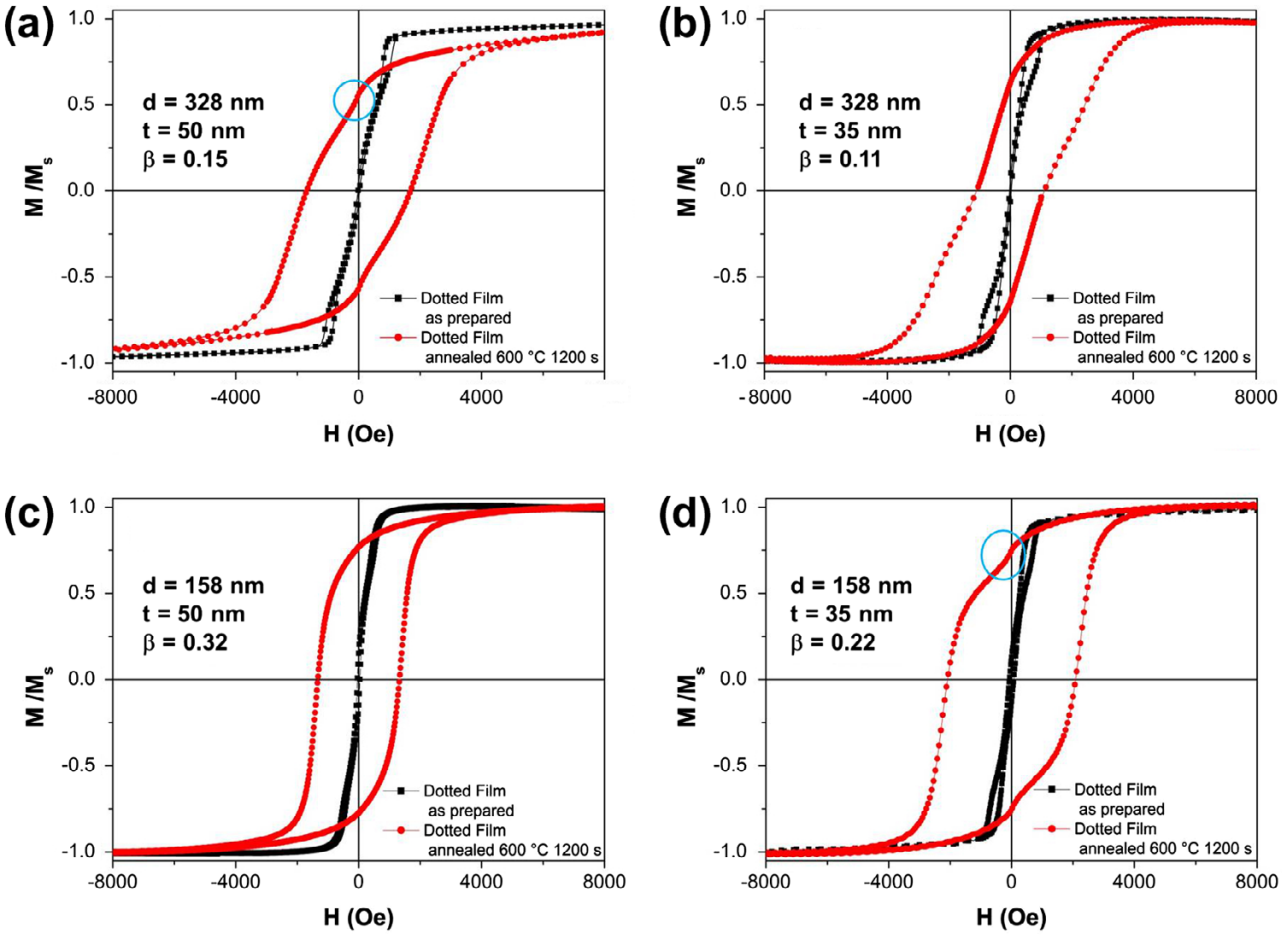
Room-temperature hysteresis loops of as-prepared and annealed (*T*_a_ = 600°C for 1200 s) Fe_50_Pd_50_ dot arrays having different aspect ratio *β*.

The results shown in Figure 7(a) and (b) are the normalized room-temperature hysteresis loops of selected annealed dotted films (*d* = 328 nm) together with the corresponding as-deposited sample measured in the parallel configuration. The disappearance of magnetization jumps corresponding to the vortex nucleation and annihilation together with a dramatic increase of coercive field mark the development of the high-anisotropy tetragonal ordered phase (see Table 2). In particular, the hysteresis curves reported are characterized by coercive field values of about 1680 Oe and 1120 Oe for thickness *t* = 50 nm and *t* = 35 nm respectively.

**Table 2.** Dot diameter (*d*); film thickness (*t*); and coercive field (*H*_c_) of annealed patterned films.

*d* (nm)	*t* (nm)	*H*_c_ (Oe)
328	50	1690
328	35	1111
158	50	1327
158	35	2084

However, the presence of a softer phase is evidenced by a magnetization kink (see circle in Figure 7(a) and (d)) occurring at low fields that can possibly indicate either the contribution of a fraction of α-Fe crystals whose presence has been confirmed by structural characterization on continuous films [21] or an incomplete transformation from A1 to L1_0_ phase promoted by the annealing. Hysteresis curves of as-prepared and annealed dotted films with *d* = 158 nm are shown in Figure 7(c) and (d) for *t* = 50 nm and *t* = 35 nm respectively. Analogously, a remarkable increase of coercivity (see Table 2) together with the disappearance of vortex annihilation and expulsion fields are observed. To extract insights about reversible and irreversible processes of the magnetization reversal mechanism, first order reversal curves have been measured on continuous and dotted films. Such an approach has been successfully used to evaluate magnetic interactions in Co nanodot array and to probe A1 to L1_0_ transformation in FePt-based thin films.

**Figure 8.**
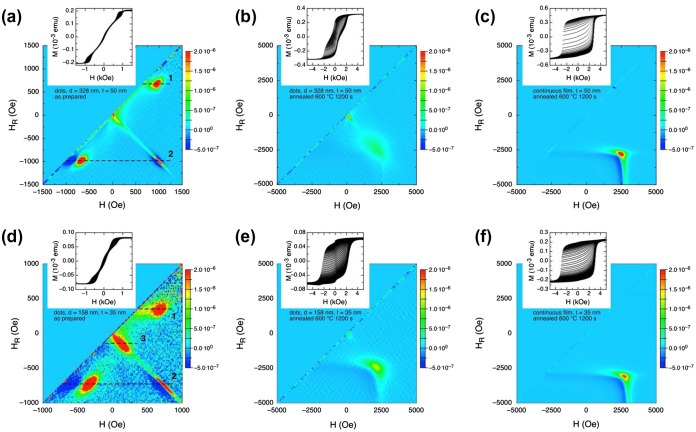
First-order reversal curves of selected Fe_50_Pd_50_ thin films: (a) patterned film with *d* = 328 nm and *t* = 50 nm; (b) annealed at *T*_a_ = 600°C for 1200 s; (c) continuous film with *t* = 50 nm, annealed at *T*_a_ = 600°C for 1200 s; (d) patterned film with *d* = 158 nm and *t* = 35 nm; (e) annealed at *T*_a_ = 600°C for 1200 s; (f) continuous film with *t* = 35 nm, annealed at *T*_a_ = 600°C for 1200 s.

In this case, after reaching saturation, the magnetization *M* is measured starting from a reversal field *H*_R_ to go back to positive saturation. FORC distribution is calculated as a mixed second order derivative *ρ*(*H*_R_, *H*) of M with respect to *H* and *H*_R_,[35] and is plotted by using a color plot (*ρ* is defined to be the intensity of the color map shown in the scale at right). For our samples, the distributions are reported in Figure 8. In addition, typical FORC curves measured at different *H*_R_ and with equal field spacing are reported in the inset of each panel in Figure 8.

In Figure 8(a), the FORC distribution of as-prepared, dotted films (*t* = 50 nm, *d* = 328 nm) allows us to obtain accurate information about nucleation and annihilation filed involved in magnetic reversal.[28,35] Along the dotted line 1 (*H*_R_ = 700 Oe), with an initial applied field of *H* = 700 Oe, vortices have already nucleated in all dots. By increasing field *H*, *ρ* increases as marked by the presence of a sharp peak at *H* = 1000 Oe. This peak corresponds to the annihilation of vortices in the dots and finally at the positive saturation *ρ* returns to zero. Instead, the line 2 fixed at *H*_R_ = –1000 Oe, starts at *H* = –1000 Oe where all dots have been negatively saturated. As *H* is increased, the first peak in the FORC distribution is at *H* = –700 Oe corresponding to the nucleation of vortices inside the dots. A second peak is seen at *H* = 1000 Oe where the vortices are annihilated. Between the two peaks, *ρ* is approximately zero, indicating that the reversible part of magnetization reversal is determined by the motion of vortices across the dots.

In Figure 8(b) and (c) the FORC distributions of the dotted and continuous films (*t* = 50 nm and *d* = 328) after the furnace treatment are shown. After annealing, the FORC distributions appear completely different because the vortex behavior disappears in favor of the appearance of a broad peak, marking a distributed irreversible mechanism responsible for the magnetization reversal occurring at large fields. Such a feature marks a distribution of anisotropy values that is observed to be wider in the annealed dot array with respect to the continuous thin film. In both annealed samples the presence of a single peak at high fields confirms a uniform development of the L1_0_ phase. In the patterned film, an additional peak at lower *H* values occurs in the color plot, confirming the presence of a softer magnetic phase as already revealed in Figure 7. The two phases clearly appear to be not interacting, as they occur in very different regions of the map. The peak corresponding to the softer phase is not evidenced in the continuous annealed film.

A similar behavior is observed in the dotted film with lower diameter (*t* = 35 nm, *d* = 158 nm) as shown in Figure 8(d). Again, along the lines 1 and 2 sharp peaks are clearly visible corresponding to the annihilation and nucleation of vortices. Additionally, in this sample a third peak is present (along line 3) marking the irreversible jump at small coercive field already observed in hysteresis loop (see Figure 6(b)).

Furnace annealed films with thickness 35 nm, either continuous or dotted, are reported in Figure 8(f) and (e) respectively. The same behavior with respect to the film having thickness *t* = 50 nm is observed, indicating that thickness variation in this range has no influence on the magnetic interactions among different phases.

**Figure 9.**
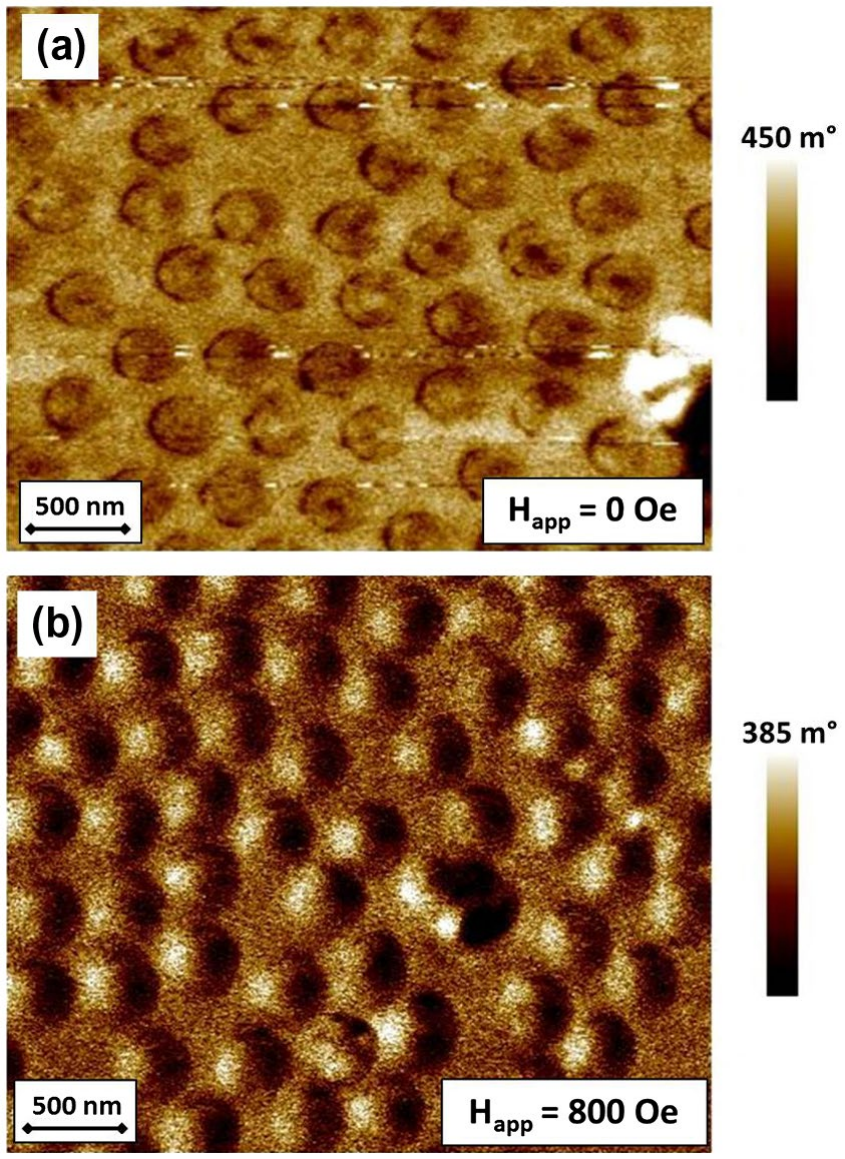
MFM images of Fe_50_Pd_50_ dot array (*d*  ≈ 328 nm, *t* = 35 nm) in different experimental conditions: (a) at magnetization remanence after applying a saturating magnetic field perpendicular to the film plane of about 10 kOe; (b) under an in-plane applied field of 800 Oe.

**Figure 10.**
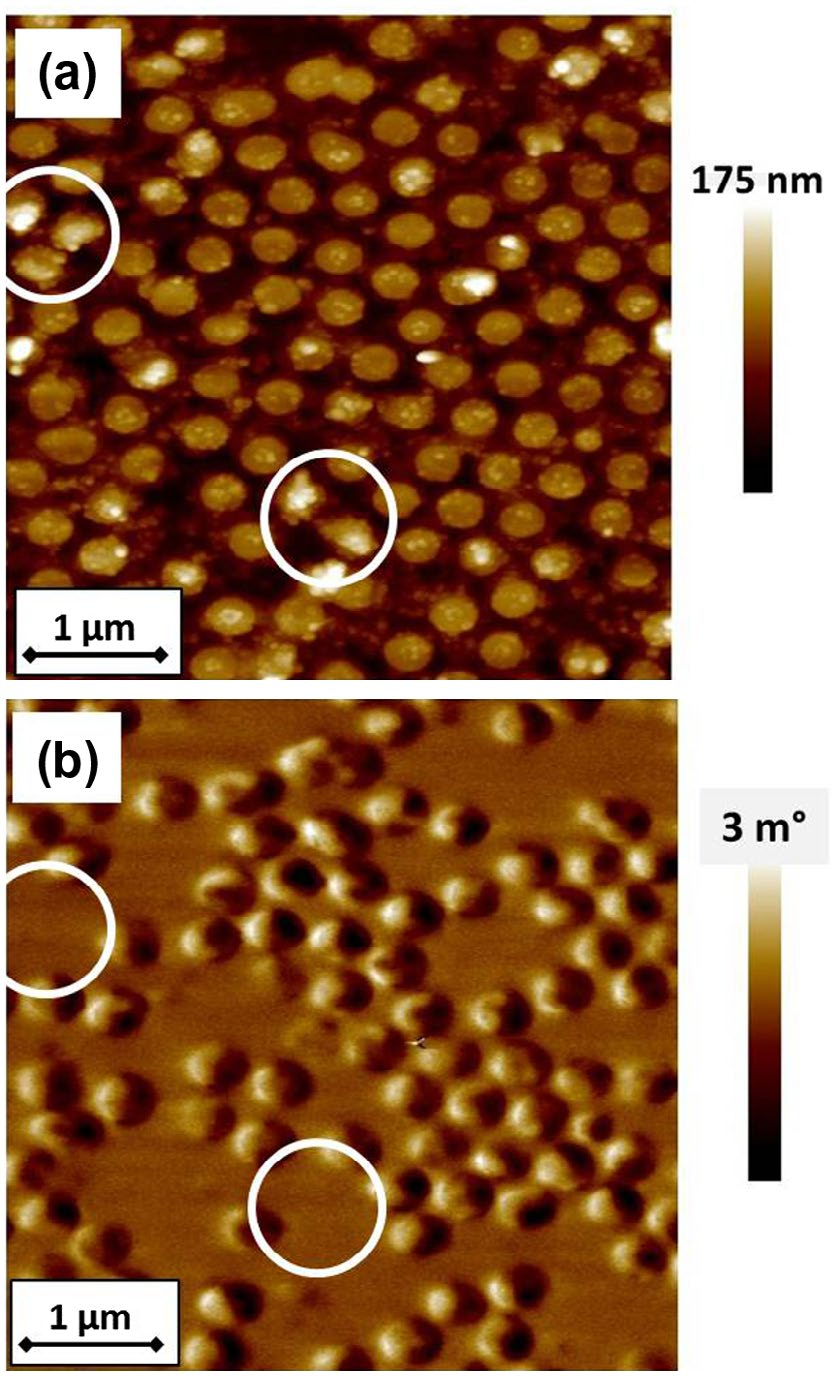
(a) AFM image of Fe_50_Pd_50_ dot array annealed at *T*_a_ = 600°C for 1200 s (*d* ≈ 328 nm, *t* = 50 nm) and (b) corresponding MFM image acquired at magnetization remanence after applying a magnetic field perpendicular to the film plane of about 10 kOe. Circles indicate different regions on the film surface.

In order to directly evidence vortex presence, magnetic force microscopy images at a remanent state are usually exploited.[16,36] As an example, an MFM image of as-prepared sample with *t* = 50 nm and *d* = 328 nm acquired at magnetization remanence after applying a magnetic field perpendicular to the film plane of about 18 kOe is reported in Figure 10(a). The magnetization reversal is controlled by a vortex process as indicated by the presence of by a darker circular region marking the vortex core in almost every dot. Such an observation is in full agreement with the hysteresis loop behavior reported in Figure 6(a). Figure 9(b) shows the magnetic domain patterns obtained applying a magnetic field of about 800 Oe in the parallel direction during the tip scan. This field value nearly corresponds to hysteresis loop region in which vortex expulsion occurs (see Figure 6(a)). Magnetic domain patterns show that the vortex configuration disappears in favor of an almost saturated state where a brighter region opposite to a darker one is visible on each dot.

The effect of annealing on the microstructure and magnetic domain patterns can characterized by AFM and MFM. To this aim, an AFM image of a furnace annealed dot array is shown in Figure 10(a) for the sample with *d* = 328 nm and *t* = 50 nm. The AFM image confirms dot size and the almost regular assembling of the PNs forming a hexagonal lattice whose regularity strictly depends on the assembling process.[24] The morphology of a few dots (see brighter regions in the circles) is altered, possibly by the development of the Pd_2_Si phase, that has been locally observed in TEM analysis (see Figure 5). In the corresponding MFM image (Figure 10(b)), as a consequence of the presence of such phase, the magnetic signal given by the corresponding dots (see circles in the figure) disappears. In the other dots, the hard L1_0_ phase is responsible for an in-plane single domain structure, that replaces the vortex configuration of the magnetically softer as-prepared dots.

## 4. Conclusions

In summary, a hierarchical self-assembling bottom-up approach based on the dispersion of a monolayer of hexagonally ordered polystyrene nanospheres on thin magnetic Fe_50_Pd_50_ films allows us to fabricate ordered 2D dot arrays. By fixing inter-dot distance and film thickness, it is possible to tune the resulting magnetization reversal process. By promoting the completion of the A1 to L1_0_ phase an enhancement of coercive field together with a different magnetization process marked by a different arrangement of magnetic domain patterns is observed. The interplay among magnetic properties, microstructure and geometrical parameters has been studied. In particular, the effect observed in key parameters of the magnetization reversal process (*H*_ann_, *H*_n_ and *H*_c_) turned out to be dependent on either aspect ratio or microstructural phases. The lack of ordering does not significantly affect functional magnetic properties, which are more influenced by geometric parameters defining dot array and the microstructural phases induced by thermal annealing. In conclusion, self-assembling still represents a simple, viable route to fabricate highly dense arrays of hexagonally close-packed magnetic nanodots over a wide area.

## Disclosure statement

No potential conflict of interest was reported by the authors.
